# Monocular meta-imaging camera sees depth

**DOI:** 10.1038/s41377-024-01666-0

**Published:** 2025-01-01

**Authors:** Yujin Liu, Xueli Chen

**Affiliations:** 1 Innovation Center for Advanced Medical Imaging and Intelligent Medicine, Guangzhou Institute of Technology, Xidian University, Guangzhou, 510555 Guangdong China; 2 Center for Biomedical-photonics and Molecular Imaging, Advanced Diagnostic-Therapy Technology and Equipment Key Laboratory of Higher Education Institutions in Shaanxi Province, School of Life Science and Technology, Xidian University, Xi’an, 710126 Shaanxi China; 3 Engineering Research Center of Molecular and Neuro Imaging, Ministry of Education & Xi’an Key Laboratory of Intelligent Sensing and Regulation of trans-Scale Life Information, School of Life Science and Technology, Xidian University, Xi’an, 710126 Shaanxi China

**Keywords:** Photonic devices, Metamaterials

## Abstract

A novel monocular depth-sensing camera based on meta-imaging sensor technology has been developed, offering more precise depth sensing with millimeter-level accuracy and enhanced robustness compared to conventional 2D and light-field cameras.

Most creatures have evolved depth perception, boosting their adaptability and survival in the environment. For humans, our binocular vision endows us with a certain depth perception ability. When looking at nearby objects, we rely on life experience to judge their distance and size; when gazing into the distance, artillerymen use the thumb method to estimate the distance to their targets, achieving precise strikes. In the TV drama “Bright Sword,” the recurring skill of thumb distance measurement by Li Yunlong has enlightened countless people.

To meet the demands of modern warfare, high-precision and motion-sensitive light detection and ranging (LiDAR) technology has been widely applied in the military domain^1^. By emitting laser pulses and measuring the time it takes for these pulses to reflect back, LiDAR technology can precisely perceive the distance and velocity of objects. As a result, LiDAR has also rapidly captured the consumer market, including applications in smart driving and robotic vacuum cleaners^2^. Despite LiDAR’s remarkable commercial success, active depth sensors that require extra controlled illumination, including ToF cameras and structured light cameras^3^, face challenges of high-power consumption and low-spatial resolution. Stereo vision systems based on multiple 2D cameras can estimates object distances through disparity that avoids the use of active lighting^4^. Nevertheless, higher precision in stereo vision systems necessitates a longer baseline between cameras, which results in greater bulk and a smaller field of view. This has prompted researchers to pursue more compact and cost-effective solutions.

Now, in a newly published paper in Light: Science & Applications^5^, Cao et al. from Tsinghua University have proposed a novel monocular depth sensing technique based a compact meta-imaging camera and an advanced analytical framework (Fig. 1). The meta-imaging camera transcends the constraints of traditional depth sensing by extracting depth data from a single viewpoint without the need for controlled illumination. It integrates a main lens, microlens array (MLA), CMOS sensor, and piezo stage into a compact, high-resolution imaging system that is resilient to optical aberrations^6^. The camera’s key innovation is its ability to overcome the spatial-angular resolution trade-off, a common challenge for light-field monocular cameras^7^. It employs digital adaptive optics to correct for multi-site aberrations, providing a significant advantage in real-world imaging scenarios.

**Fig. 1:**
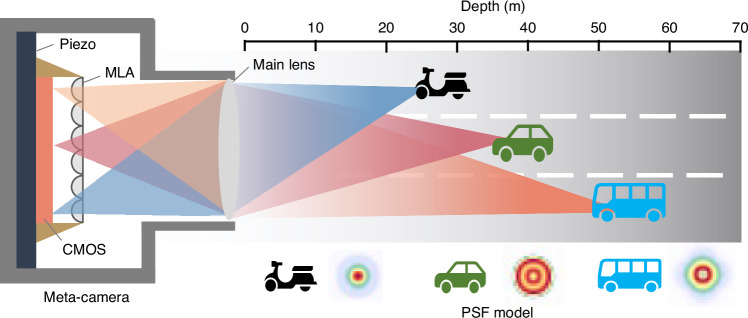
Schematic view of the monocular meta-imaging camera that can precisely sense depth by a straightforward point spread functions (PSF)-based depth estimation method

The meta-imaging camera’s precision is quantified through the computation of the Cramér–Rao lower bound (CRLB), and its theoretical precision is validated with simulations and experiments, showing superior performance across a broader depth range and excellent robustness against changes in signal-background-ratio (SBR). The meta-imaging camera’s ability to achieve millimeter-level accuracy in depth estimation, even in the presence of optical aberrations, is particularly noteworthy, making it crucial for applications where depth information is essential for safe and effective operation.

This camera’s integration of microlens into a depth-sensing system marks a significant advancement, offering higher precision and robustness in depth perception compared to conventional imaging technologies. The meta-imaging camera’s breakthrough technology enhances monocular depth sensing with its compact design, making it suitable for a range of applications from augmented reality to autonomous vehicles, where precise depth perception is crucial for seamless digital overlays and safe navigation. Its potential extends to environmental monitoring and medical imaging, promising high-precision depth sensing even under challenging conditions.
